# Comparison of open and percutaneous navigated pedicle screw placement for traumatic injuries of the subaxial cervical spine

**DOI:** 10.1016/j.bas.2025.105880

**Published:** 2025-11-17

**Authors:** Fenna Brunken, Jula Gierse, Philipp Raisch, Paul A. Grützner, Sven Y. Vetter

**Affiliations:** BG Klinik Ludwigshafen, Department for Orthopaedics and Trauma Surgery at Heidelberg University, Ludwig-Guttmann-Str. 13, 67071, Ludwigshafen, Germany

**Keywords:** Cervical pedicle screw placement, Trauma, Spinal navigation, Subaxial spine injury

## Abstract

**Introduction:**

Cervical pedicle screw (CPS) placement is a technically demanding procedure with a high risk of neurovascular injuries in case of screw misplacement. In contrast to the thoracolumbar spine, there is limited data on percutaneous pedicle screw placement in the cervical spine.

**Research question:**

This study aims to compare the accuracy, blood loss and duration of surgery for open and percutaneous CPS placement for traumatic injuries of the subaxial cervical spine.

**Materials and methods:**

In this retrospective single-center study, 22 patients undergoing percutaneous CPS placement were compared to a control group of 18 patients treated with open CPS placement. The screw position was analyzed in postoperative CT scans using the Neo and Bredow classifications. Additionally, intraoperative radiation exposure, duration of surgery, and blood loss were evaluated.

**Results:**

The overall accuracy (perforation <2 mm) was 89.71 % for percutaneous and 89.29 % for open CPS placement (p > 0.99). None of the misplaced screws resulted in neurovascular injuries. One patient in the control group underwent revision surgery due to screw misplacement. The mean duration of surgery and intraoperative blood loss were lower in percutaneous compared to open procedures. There was no significant difference in intraoperative radiation exposure between the two groups.

**Discussion and conclusion:**

The results suggest that percutaneous CPS placement in the subaxial spine is feasible with similar accuracy compared to open CPS placement. Further studies are needed to evaluate long-term clinical outcomes.

## Highlights

• Percutaneous cervical pedicle screw placement is feasible in subaxial spine trauma.
• Accuracy was similar for open and percutaneous cervical pedicle screw placement.
• Blood loss and duration of surgery were reduced in percutaneous procedures.
• Radiation exposure did not differ significantly between both techniques.

## 1 Introduction

Dorsal instrumentation of the cervical spine is used for the treatment of various conditions, including traumatic injuries, degenerative disorders or neoplastic diseases. Previous studies have demonstrated that cervical pedicle screw (CPS) fixation provides superior mechanical stability compared to lateral mass screw fixation (LMS) (Johnston et al., 2006; Jones et al., 1997; Ito et al., 2014; Kothe et al., 2004).

However, pedicle screw placement in the cervical spine is considered more difficult compared to other spinal regions due to smaller pedicle diameters and the proximity of critical structures such as the spinal canal, nerve roots and the vertebral artery (Soliman et al., 2022; Yoshihara et al., 2013; Abumi et al., 2000; Onibokun et al., 2009). Accordingly, a high accuracy is necessary in CPS placement to minimize the risk of neurovascular injuries.

Due to the angulation of pedicles in the cervical spine, conventional techniques require a wide incision and extensive exposure to visualize the entry points, often resulting in significant soft-tissue trauma (Mohi, 2014; Zhou et al., 2017). Minimally invasive techniques have gained popularity in spine surgery for different indications. In the thoracolumbar spine, percutaneous pedicle screw placement has been shown to reduce paraspinal tissue trauma, decrease blood loss and shorten recovery time (Jiang et al., 2012; Vanek et al., 2014).

Although percutaneous pedicle screw placement may offer similar advantages in the cervical spine, it is less frequently performed. Percutaneous pedicle screw placement relies on precise intraoperative imaging due to reduced visual control. The lower cervical spine can be difficult to visualize using conventional fluoroscopy due to the superimposition of the patient's shoulders. Previous studies have reported high accuracy rates for navigated CPS placement using intraoperative computed tomography (iCT), particularly in traumatic injuries (Bertram et al., 2021; Habib et al., 2021). However, there is limited clinical data on the accuracy of percutaneous CPS placement.

This study aims to compare the accuracy of navigated percutaneous and open CPS placement for the treatment of subaxial cervical spine injuries.

## 2 Materials and Methods

In this single-center retrospective study, at a level I trauma center, 22 patients undergoing percutaneous pedicle screw placement at the subaxial cervical spine were included consecutively from July 2023 to May 2025. The results were compared to a control group of all patients treated with open CPS placement between April 2023 and February 2025 at the same institution. Open or percutaneous CPS placement was performed at the surgeon's discretion, mainly depending on the need for dorsal decompression, which was only performed in the open group. The surgeries were performed by three spine surgeons, with both open and percutaneous procedures included for each surgeon.

Only patients with traumatic injuries (fractures, osteoligamentous and ligamentous distraction injuries) of the subaxial cervical spine (C3-7) and available postoperative CT scans were included. Patient consent was waived by the local ethics committee (application number: 2025–18051).

### 2.1 Surgical technique

Patients were placed in prone position on a radiolucent carbon fiber surgical table with the head fixed in a Mayfield clamp. The correct spinal levels were identified using fluoroscopy.

**Fig. 1:**
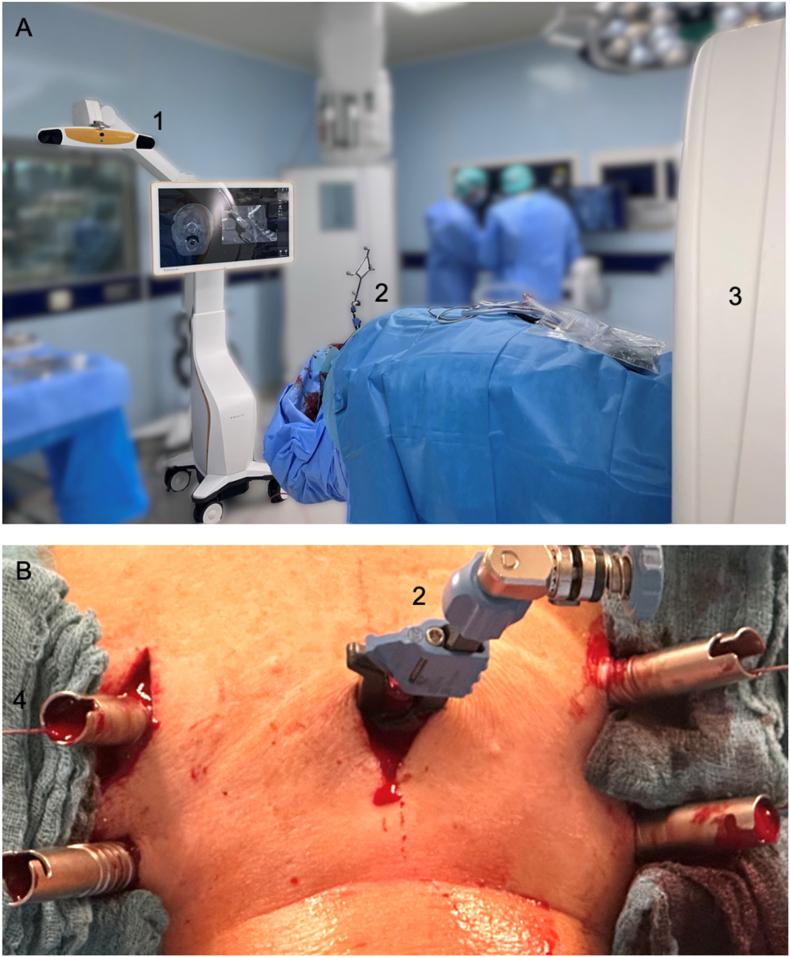
A: Intraoperative setup for navigated CPS placement showing the positions of the camera (1), patient reference array (2) and iCT (3). B: Example of percutaneous CPS placement at C4-6 showing the patient reference array (2) and guidewires (4).

For open procedures the entry points were visualized using a standard midline approach. In percutaneous procedures, a small midline skin incision was made to place the patient reference array (Fig. 1). In both groups the patient reference array was mounted on a spinous process in the middle of the surgical field to avoid a distance of more than two segments to the vertebrae to be instrumented. A registration scan was acquired by iCT (Airo, Stryker, Kalamazoo, US) or C-arm cone beam computed tomography (CBCT) (Cios Spin, Siemens Healthineers, Erlangen, Germany) and automatically transferred to the navigation platform (Curve Image Guided Surgery, Brainlab, Munich, Germany). All pedicle screws were placed using intraoperative navigation. A navigated probe was used to determine the correct entry point. For percutaneous pedicle screw placement additional paramedian stab incisions were made for each screw and the navigated drill guide was inserted through the incisions. The trajectory was continuously visualized on the navigation platform. After drilling, guidewires were placed through the drill guide and the position was verified by standard fluoroscopy. Screw sizes were initially planned based on preoperative CT and adapted intraoperatively if necessary. The screw positions were controlled by standard fluoroscopy and additional intraoperative 3D imaging using iCT or CBCT was performed at the surgeon's discretion. Finally, the rods were inserted and secured to the screws. The same implant system was used in all cases (Ennovate Cervical, B. Braun, Melsungen, Germany). A postoperative CT was performed two days after surgery.

### 2.2 Data acquisition

Patient demographic data, operative time (min) and postoperative complications were retrieved from the electronic patient chart. The dose report was used to collect intraoperative fluoroscopy time (s), intraoperative dose area product (DAP) (mGy∗cm^2^) and dose-length product (DLP) (mGy∗cm).

Screw perforations were measured in multiplanar reconstructions of postoperative CT scans (0.5 mm slices) and screw accuracy was assessed according to the Neo and Bredow classification (Criteria shown in Table 1, Table 2) (Bredow et al., 2016; Neo et al., 2005). Each screw was measured twice by the same observer, blinded to the surgical approach and clinical outcomes, and the mean perforation value was used for further assessment.

**Table 1:** Definition of the Neo classification.

Neo Classification	
Grade	Definition
0	no perforation
1	perforation <2 mm
2	perforation ≥2 and < 4 mm
3	perforation ≥4 mm

**Table 2:** Definition of the Bredow classification.

Bredow Classification	
Grade	Definition
1	perforation <1 mm
2	perforation <2 mm
3	perforation <3 mm
4	perforation <4 mm
5	perforation ≥4 mm and/or obstruction of transverse foramen by over half a screw diameter

Critical perforations were defined as perforations of ≥2 mm and/or obstruction of the transverse foramen by more than half a screw diameter, corresponding to Neo grades 2 and 3 and Bredow grades 3–5. Additionally, the pedicle width at the instrumented levels was measured in preoperative CT scans to calculate the screw diameter to pedicle width (SD/PW) ratio for each screw.

### 2.3 Statistical analysis

Data were analyzed using Prism 10 (GraphPad, San Diego, USA) and R 4.3.2 (R Core Team, Vienna, Austria). In order to improve comparability, cases involving laminectomies, surgery on additional spinal regions, or placement of more than four screws were excluded from the analysis of surgery time and blood loss. Shapiro-Wilk test was used to assess normality of data. An unpaired *t*-test was applied for the comparison of two groups for normally distributed data. For the comparison of non-parametric data, the Mann-Whitney test was used. For multiple comparisons of normally distributed data, an ANOVA with Tukey's post-hoc correction was used. Fisher's exact test was used for the analysis of nominal data. Descriptive statistics are presented as means ± standard deviation for continuous variables and absolute numbers with percentages for categorical variables. Intra-rater reliability for the measurement of screw perforations was assessed using the intraclass-correlation coefficient (ICC) based on a two-way mixed effects model for absolute agreement of single measurements. ICC was interpreted according to Cicchetti's recommendations (Cicchetti, 1994). P-values <0.05 were considered statistically significant.

## 3 Results

In total, 22 patients undergoing percutaneous cervical pedicle screw placement were compared to 18 patients treated with open cervical pedicle screw placement. There were no significant differences regarding demographic data or procedure characteristics between the two groups (see Table 3).

**Table 3:** Demographic data and procedure characteristics.

	Open		Percutaneous		p
Mean age (years)	68.11 ± 17.73		67.50 ± 22.28		*0.67*^a^
BMI (kg/m^2^)	27.22 ± 6.11		26.22 ± 3.87		*0.54*^b^
	n	%	n	%	
Gender					*0.72*^c^
Male	14	(77.78)	15	(68.18)	
Female	4	(22.22)	7	(31.81)	
AO Classification					*0.85*^c^
A4	0	(0.0)	1	(4.55)	
B1	3	(15.79)	2	(9.09)	
B2	3	(15.79)	6	(27.27)	
B3	8	(42.11)	9	(40.91)	
C	5	(26.32)	4	(18.18)	
Instrumented vertebrae					*0.16*^c^
C3	3	(10.71)	0	(0.0)	
C4	5	(17.86)	6	(17.65)	
C5	6	(21.43)	10	(29.41)	
C6	6	(21.43)	13	(38.24)	
C7	8	(28.57)	5	(14.71)	
Number of screws					*0.35*^c^
4	14		20		
6	2		2		
8	2		0		

a) Mann-Whitney test.

b) Unpaired *t*-test.

c) Fisher's exact test.

A total of 68 screws placed from C3 to C7 were analyzed in the percutaneous group, while 56 screws were assessed in the control group.

In the percutaneous group, ventral fusion was performed in five cases prior to dorsal instrumentation. In the control group, ventral fusion was performed in three cases prior to and three cases after dorsal instrumentation. Laminectomies were performed in eleven cases in the control group.

**Fig. 2:**
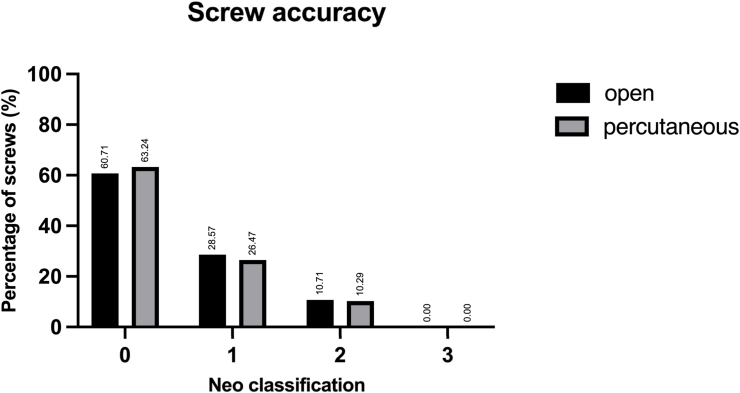
Screw placement accuracy according to the Neo classification for both groups.

**Fig. 3:**
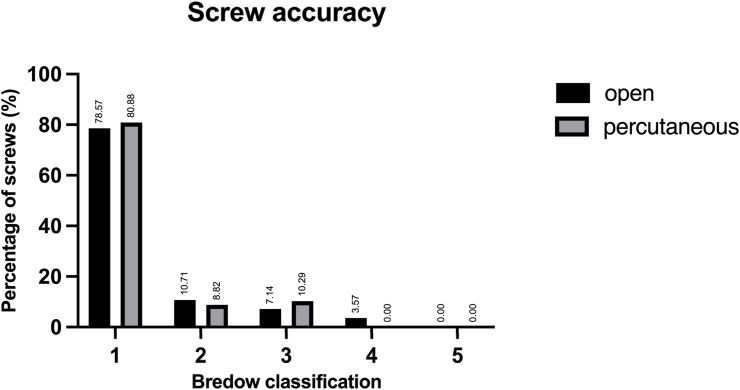
Screw placement accuracy according to the Bredow classification for both groups.

Overall, 89.71 % of screws in the percutaneous group were classified as accurate (Neo grade 0–1 or Bredow grade 1–2), compared to 89.29 % in the open group (p > 0.99) (see Fig. 2, Fig. 3).

None of the screws in the percutaneous group was rated Neo grade 3 or Bredow grades 4–5. In the control group, two screws were rated Bredow grade 4. None of the pedicle perforations resulted in neurovascular deficits. In one case in the control group revision surgery was performed due to screw misplacement (Bredow grade 4), while no revision surgeries were required in the percutaneous group. Additionally, a wound healing disorder requiring surgical revision was observed in one case in the open group.

The most common direction of perforation was medial (48.94 %), followed by lateral (40.43 %) and inferior (10.64 %) perforations.

The intraclass correlation coefficient (ICC) for intra-observer agreement of screw perforation measurements was 0.96 (95 % CI 0.94–0.97), indicating a good agreement between the measurements.

**Fig. 4:**
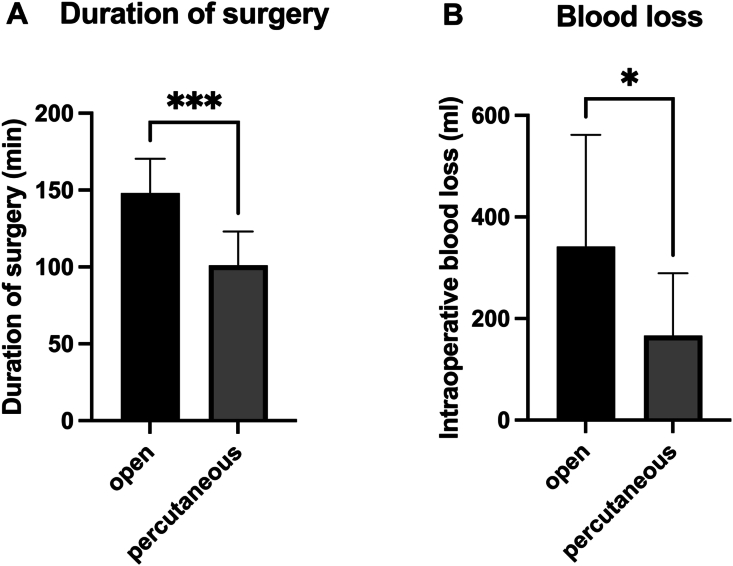
A: Mean duration of surgery for open and percutaneous procedures. B: Mean intraoperative blood loss for both groups. Data are displayed as means ± standard deviation. ∗p < 0.05.∗∗∗p < 0.001.

The mean duration of surgery was 101.1 ± 22.10 min in the percutaneous group and 148.0 ± 22.41 min in the control group (p = 0.0002). The mean intraoperative blood loss was lower for percutaneous (166.4 ± 122.9 ml) compared to open procedures (341.7 ± 220.0 ml) (p = 0.049) (see Fig. 4).

There was no significant difference in intraoperative radiation exposure from iCT (DLP) or conventional fluoroscopy and CBCT (DAP) between the two groups. The mean DAP was slightly higher in the open group (6618 ± 8344 mGy∗cm^2^) compared to the percutaneous group (5964 ± 6288 mGy∗cm^2^) (p = 0.95) while the mean DLP was lower in the open group (384.8 ± 67.70 mGy∗cm) compared to the percutaneous group (437.5 ± 87.48 mGy∗cm) (p = 0.35). The mean fluoroscopy time was 66.71 ± 44.83 s for open and 67.29 ± 40.11 s for percutaneous procedures (p = 0.78) (see Table 4).

**Table 4:** Intraoperative radiation exposure.

	Open	Percutaneous	p
DAP (mGy∗cm^2^)	6618 ± 8344	5964 ± 6288	*0.95*^a^
DLP (mGy∗cm)	384.8 ± 67.70	437.5 ± 87.48	*0.35*^b^
Fluoroscopy time (s)	66.71 ± 44.83	67.29 ± 40.11	*0.78*^a^

Abbreviations: DAP: dose area product; DLP: dose length product.

a) Mann-Whitney test.

b) Unpaired *t*-test.

**Fig. 5:**
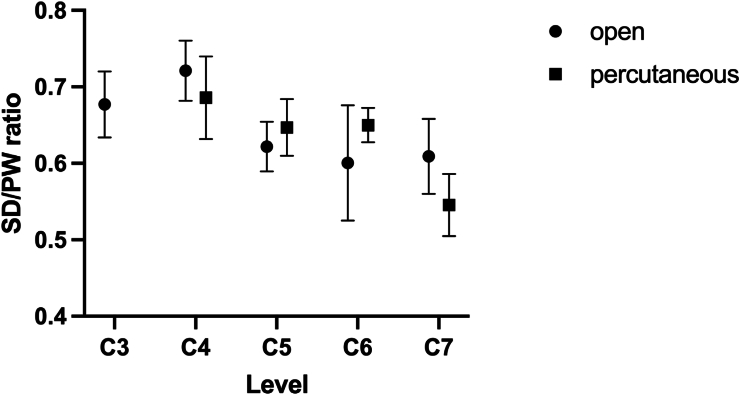
SD/PW ratios for different spinal levels for open and percutaneous CPS placement.

There was no significant difference in SD/PW ratios between open and percutaneous CPS placement across all vertebral levels. Overall, the mean SD/PW ratio was 0.64 ± 0.09 for the open group and 0.64 ± 0.08 for the percutaneous group (p = 0.81). No significant difference in pedicle widths was observed between the two groups. The overall SD/PW ratio was significantly higher at C4 compared to C5, C6 and C7 (all p < 0.05). SD/PW ratios are shown in Fig. 5.

## 4 Discussion

Cervical pedicle screws were first introduced by Abumi et al. in 1994 (Abumi et al., 1994). Although it has been shown that CPS are biomechanically superior to LMS, their use has been limited due to technical demands and concerns about potential neurovascular injuries (Johnston et al., 2006; Jones et al., 1997; Ito et al., 2014; Kothe et al., 2004). Misplacement rates of 6.7–27 % have been reported for CPS placement using standard fluoroscopy (Abumi et al., 2000; Hojo et al., 2014; Ishikawa et al., 2010). Visualization of the lower cervical spine by conventional fluoroscopy can be complicated by the superimposition of the patient's shoulders in lateral views. Lately, intraoperative 3D imaging and navigation systems have gained popularity in spine surgery for different indications.

Previous studies have demonstrated a high accuracy for open navigated CPS placement, with reported accuracy rates ranging between 78 and 99 % in the subaxial spine (Habib et al., 2021; Bindels et al., 2024; Bredow et al., 2015; Barsa et al., 2016). However, two recent meta-analyses did not find a significant difference in accuracy between navigated and non-navigated subaxial CPS placement (Bindels et al., 2024; Zhou et al., 2024).

While several studies have evaluated the accuracy of CPS placement using an open approach, there is limited comparable data for percutaneous placement of pedicle screws in the subaxial cervical spine. Due to limited visual control, reliable intraoperative imaging is necessary to safely perform percutaneous pedicle screw placement. Schaefer et al. reported that 42.11 % of pedicle screws placed percutaneously in the subaxial cervical spine without navigation showed perforations of >1 mm (Schaefer et al., 2011). Our study demonstrated a higher accuracy for navigated CPS placement with perforations >1 mm in 17.65 % of screws in the percutaneous and 21.43 % in the open group. A cadaveric study found an accuracy rate of 89.8 % (Bredow grade 1–2) for navigated percutaneous CPS placement from C3-7, which is similar to the accuracy of 89.71 % for percutaneous CPS placement observed in this study (Schmeiser et al., 2025). In a recent study Blume et al. also reported an accuracy of 90 % for navigated percutaneous CPS placement (Blume et al., 2025).

Comparing the accuracy of CPS across previous studies is complicated due to the use of different grading systems, including the Gertzbein-Robbins, Neo, and Bredow classifications (Bertram et al., 2021; Bredow et al., 2016; Neo et al., 2005; Richter et al., 2005; Chachan et al., 2018)*.* To allow comparability with existing literature we decided to report the two most commonly used classifications (Neo and Bredow classification). Since no obstruction of the transverse foramen by more than half a screw diameter (Bredow 5) was observed in the current study, the proportion of accurate screws defined as Neo 0–1/Bredow 1–2 was identical.

It has to be noted that the high mobility of the cervical spine in general and especially in unstable injuries might affect the accuracy of intraoperative navigation due to changes in anatomy after acquisition of the registration scan. In the current study, only patients with traumatic injuries of the cervical spine were included, which should be considered when comparing the accuracy with previous studies that included patients with various indications for CPS placement. Bertram et al. reported a similar accuracy of 88 % for open CPS placement (C2-7) in trauma patients using iCT-based navigation (Bertram et al., 2021). To minimize navigation inaccuracies, excessive pressure during screw placement should be avoided and navigation accuracy should be verified regularly on different anatomic landmarks using the navigation pointer. Interestingly, it has been shown that the distance from the reference array does not influence the accuracy of percutaneous CPS placement from C3 to C7 when the reference array is placed on C7 in cadaveric models with intact cervical spines (Schmeiser et al., 2025).

Screw misplacement in the cervical spine can lead to serious sequelae. However, despite the narrow corridor for safe pedicle screw placement in the cervical spine, neurovascular injuries do not occur frequently. The incidence of vertebral artery injuries after CPS placement reported in the literature is low, ranging from 0.2 to 0.61 %, even with lateral perforations >2 mm (Soliman et al., 2022; Yoshihara et al., 2013; Abumi et al., 2000; Schaefer et al., 2011).

According to literature the most common direction of perforation in CPS is lateral (Schaefer et al., 2011; Yoshimoto et al., 2009; Lee et al., 2017). In the current study medial perforations (48.94 %) occurred slightly more frequently than lateral (40.43 %). The relative reduction in lateral misplacements might be explained by the use of additional lateral stab incisions reducing the lateral pressure from paravertebral muscles (Shimokawa and Takami, 2017).

Screw misplacement in the sagittal plane, particularly cranial perforations, can violate the neural foramen and cause injuries of the nerve roots (Abumi et al., 2000; JUNG et al., 2020; Ghori et al.). In a case series of 27 patients undergoing navigated minimally invasive CPS fixation, one patient required revision surgery due to a malpositioned screw leading to radiculopathy (Coric and Rossi, 2022a, 2022b). In the current study, no neurovascular injuries were observed. However, revision surgery was performed in one patient in the open group due to screw misplacement (Bredow Grade 4).

Several studies have shown that percutaneous pedicle screw placement in the thoracolumbar spine is highly accurate and leads to reduced paraspinal muscle trauma, lower blood loss and shorter recovery times (Jiang et al., 2012; Vanek et al., 2014). Due to the angulation of cervical pedicles, a wide exposure is needed to visualize the entry points using a standard midline approach. Percutaneous CPS placement aims to reduce the risk of wound healing complications, infection rates and postoperative muscle atrophy due to approach-related soft-tissue trauma.

For open CPS fixation, wound healing complications have been observed in up to 10.2 % of patients and revision surgery for postoperative infections was required in up to 2.52 % of patients (Bredow et al., 2015; Lee et al., 2017). In the current study a wound healing disorder requiring surgical revision was observed after open CPS placement in one patient.

Another benefit of percutaneous pedicle screw placement might be a reduction of intraoperative blood loss. In line with previous reports the mean blood loss was lower in percutaneous compared to open procedures. Sugimoto et al. reported a blood loss of 780 ml for open CPS placement compared to 180 ml for minimally invasive CPS placement (Sugimoto et al., 2017). The mean blood loss for open CPS placement was lower in the current study, however it should be noted that patients undergoing laminectomy were excluded from the analysis in this study to improve comparability to the percutaneous group (Sugimoto et al., 2017). Additionally, the mean duration of surgery was also lower in the percutaneous group compared to the control group.

While the effect of the SD/PW ratio on biomechanical stability has been investigated in several studies in the thoracolumbar spine, clinical or biomechanic data for cervical pedicle screws are lacking (Matsukawa et al., 2021; Solitro et al., 2024). In a sawbone model, SD/PW ratios of 0.64 at C7 to 1.10 at C4 have been observed (Mandelka et al., 2024). Overall, SD/PW ratios were lower in the current study. In line with the previous study, the lowest SD/PW ratio was also found at C7 (Mandelka et al., 2024). Typically screw diameters of 3.5, 4.0 or 4.5 mm are used at the cervical spine. In this study, the average pedicle widths ranged from 5.23 ± 0.55 mm at C4 to 6.53 ± 0.83 mm at C7, which is comparable to previous data reporting pedicle widths between 5.0 ± 1 mm at C4 and 6.5 ± 1.2 mm at C7 (Onibokun et al., 2009).

There was no difference in SD/PW ratios between open and percutaneous approach probably due to the use of intraoperative navigation in both groups.

In the current study there was no significant difference in intraoperative radiation exposure between open and percutaneous pedicle screw placement. In both groups a reference scan for navigation was acquired by iCT or CBCT. Additional intraoperative 3D scans were performed at the surgeon's discretion. There was no significant difference in the number of additional 3D scans between open and percutaneous CPS placement. To reduce radiation exposure, C-arm CBCT was preferentially used in this study if intraoperative 3D imaging was required to verify screw positioning (Mandelka et al., 2024; Klingler et al., 2022). In both cases, OR personnel leave the room during scan acquisition to limit the staff radiation exposure. Despite the potential benefits it has to be noted that a 3D C-arm CBCT and iCT may not be readily available in all clinical settings at the same time. Previous studies suggest that CBCT-based navigation may be a similarly accurate alternative to iCT-based navigation (Mandelka et al., 2024; Kendlbacher et al., 2022).

### 4.1 Limitations

Since this is single-center retrospective study several limitations have to be considered. Although no statistically significant difference in accuracy was observed between open and percutaneous CPS placement, the small sample size limits the statistical power and the ability to detect small differences between groups. As this is a retrospective study no formal clinical follow-up was conducted. Further prospective studies are needed to compare the long-term clinical outcome of percutaneous and open CPS placement. In addition, the indications for percutaneous CPS fixation remain narrow, limiting its use in traumatic cervical injuries to cases without the need for dorsal decompression or following anterior decompression and fixation. In the current study, laminectomies were performed exclusively in the open group and were carried out after screw placement. To ensure comparability between the open and percutaneous groups, cases involving laminectomies were excluded from the analysis of operative time and intraoperative blood loss.

## 5 Conclusion

The results of this study indicate that navigated percutaneous placement of pedicle screws in the subaxial spine is feasible with similar accuracy compared to open CPS placement. Further prospective studies are needed to evaluate the long-term clinical outcome of percutaneous CPS fixation without an additional fusion.

## Ethical approval and informed consent statements

This study was reviewed by the responsible ethics committee (Rhineland-Palatinate Medical Association, application number 2025–18051, date: March 24, 2025). Patient consent was waived due to the retrospective analysis of anonymized patient data. All procedures were in accordance with the ethical standards of the institutional and national research committee and with the 1964 Helsinki declaration and its later amendments or comparable ethical standards.

## Data availability statement

The data that support the findings of this study are available from the corresponding author on reasonable request.

## Declaration of generative AI and AI-assisted technologies in the writing process

During the preparation of this work the authors used ChatGPT-4 (OpenAI, San Francisco, California, USA) and Grammarly (Grammarly Inc,. San Francisco, California, USA) in order to check grammar and spelling. After using this tool/service, the authors reviewed and edited the content as needed and take full responsibility for the content of the publication.

## Funding

The author(s) did not receive any specific financial support from funding agencies in the public, commercial, or not-for-profit sectors for the research, authorship, and/or publication of this article.

## Declaration of competing interest

The authors declare the following financial interests/personal relationships which may be considered as potential competing interests:The research group MINTOS received grants/has grants pending and technical support from Siemens Healthineers AG (Erlangen, Germany) and Globus Medical Inc. (Audubon, Pennsylvania, USA). The funders had no involvement in the study conceptualization, design, data collection, analysis, writing of the manuscript, or decision to submit the manuscript for publication.
